# Not All Grams per Deciliter of Hemoglobin Are Equal

**DOI:** 10.1016/j.jscai.2023.101229

**Published:** 2023-11-27

**Authors:** Zach Rozenbaum

**Affiliations:** Department of Cardiology, Tulane University, New Orleans, Louisiana

**Keywords:** bleeding, pulmonary embolism, pulmonary thrombectomy, thrombolysis

In the January 2024 issue of *JSCAI*, Monteleone et al^1^ compared the bleeding risk of ultrasound-assisted catheter-directed thrombolysis (USCDT) for pulmonary embolism vs mechanical thrombectomy (MT). The study found that USCDT was associated with a lower risk of bleeding. Importantly, these results represent an epidemiologic perspective and do not integrate patient-level care. There are practical factors to be considered when comparing the 2 therapies.

First, procedural blood loss during MT is generally controlled unless it results from vascular complications which are relatively uncommon. Despite the practice of blood filtering and returning to the patient during MT, certain procedural blood loss is expected. Typically, a decrease in hemoglobin is only observed 12 to 24 hours after the procedure due to several reasons, such as hemodilution. The decrease in hemoglobin is predictable and does not represent bleeding. Therefore, not all decreases in grams per deciliter of hemoglobin are equal and necessarily represent a complication.

Second, when bleeding occurs during USCDT, it occurs under thrombolytic therapy. Although most sources of bleeding are access site related, active hemorrhage in patients who receive thrombolytic therapy is not equivalent to hemorrhage in anticoagulated patients. Moreover, heparinization is monitored and may be effectively and swiftly reversed versus thrombolysis.

Another important factor to be taken into account when evaluating outcomes, especially other than bleeding, in the current study is the lack of risk stratification. Unstable patients are less likely to be treated with USCDT because of the length of treatment. MT achieves immediate results if successful, and consequently inherently includes more unstable patients.

The study provides important information. However, all aspects of the results should be taken into account during their interpretation.
